# Crystal structure, Hirshfeld surface analysis and DFT studies of tetra­kis­(μ-3-nitro­benzoato-κ^2^ *O* ^1^:*O* ^1′^)bis­[(*N*,*N*-di­methyl­formamide-κ*O*)copper(II)] di­methyl­formamide disolvate

**DOI:** 10.1107/S2056989021010999

**Published:** 2021-10-26

**Authors:** Mavlonbek A. Ziyaev, Jamshid M. Ashurov, Alisher G. Eshimbetov, Bakhtiyar T. Ibragimov

**Affiliations:** a Institute of Bioorganic Chemistry, Uzbekistan Academy of Sciences, 100125, Mirzo Ulugbek Str.,83, Tashkent, Uzbekistan

**Keywords:** crystal structure, binuclear copper complex, DFT calculations, di­methyl­formamide, FT–IR spectroscopy, 3-nitro­benzoate

## Abstract

The title compound, [Cu_2_(C_7_H_4_NO_4_)_4_(C_3_H_7_NO)_2_]·(C_3_H_7_NO)_2_, is a binuclear copper(II) complex located on an inversion center midway between the two copper(II) cations. The asymmetric unit consists of one Cu^II^ cation, two 3-nitro­benzoato ligands, and two di­methyl­formamide (DMF) mol­ecules, one of which coordinates to the Cu^II^ cation and one is a solvate mol­ecule. The carboxyl­ate groups of the ligands bridge two Cu^II^ cations with a Cu—Cu distance of 2.6554 (6) Å, completing a distorted octa­hedral O_5_Cu coordination environment. The dihedral angles between the carboxyl­ate and the aromatic ring planes of the two independent ligands are different from one another, *viz*. 5.2 (3) and 23.9 (3)°. The three-dimensional structure is consolidated by weak C—H⋯O inter­actions and stabilized by π–π stacking inter­actions between the aromatic rings. The complex and the free ligand were further characterized by Fourier-transform infrared spectroscopy (FT–IR), and the energies of the frontier mol­ecular orbitals of the complex were determined by DFT calculations at the B3LYP/def2-TZVP level of theory.

## Abstract 2

The title compound, [Cu_2_(C_7_H_4_NO_4_)_4_(C_3_H_7_NO)_2_]·(C_3_H_7_NO)_2_, is a binuclear copper(II) complex located on an inversion center midway between the two copper(II) cations. The asymmetric unit consists of one Cu^II^ cation, two 3-nitro­benzoato ligands, and two di­methyl­formamide (DMF) mol­ecules, one of which coordinates to the Cu^II^ cation and one is a solvate mol­ecule. The carboxyl­ate groups of the ligands bridge two Cu^II^ cations, completing a distorted octa­hedral O_5_Cu coordination environment.

## Chemical context

Copper complexes have been explored extensively due to the fact that copper is a bio-essential element responsible for numerous bioactivities in living organisms (Tapiero & Tew, 2003 ▸). Moreover, it is well known that Cu^II^ complexation plays an important role in the pharmacological profile of anti­microbial activities (Haiduc & Silvestru, 1989 ▸; Linder & Goode, 1991 ▸). The first *syn–syn* bridged binuclear structure of a large number of copper(II) carboxyl­ates with general formula [Cu(*R*COO)_2_(*L*)]_2_ (*L* = co-ligand), was reported for simple copper(II) acetate monohydrate (Van Niekerk & Schoening, 1953 ▸). This classical structure consists of a binuclear [Cu_2_O_8_] unit in which each copper(II) atom is surrounded by four oxygen atoms of carboxyl­ate groups in an almost square-planar coordination. An additional ligand, here the O atom of a water mol­ecule, is attached in an apical position at longer Cu—O distances. The Cu—Cu contact completes a distorted octa­hedral coordination sphere around each copper(II) atom. This motif is also observed in polymeric copper(II) carboxyl­ates, where the apical ligand has two coordination centers and links dimeric units (Rao *et al.*, 1983 ▸; Zhu *et al.*, 2003 ▸). In the situation where the apical ligand is absent, a zigzag polymeric structure is formed with direct bonding between [Cu_2_O_8_] units *via* the metal and one of the basal oxygen atoms of the neighbouring unit (Drożdżewski *et al.*, 2004 ▸). 
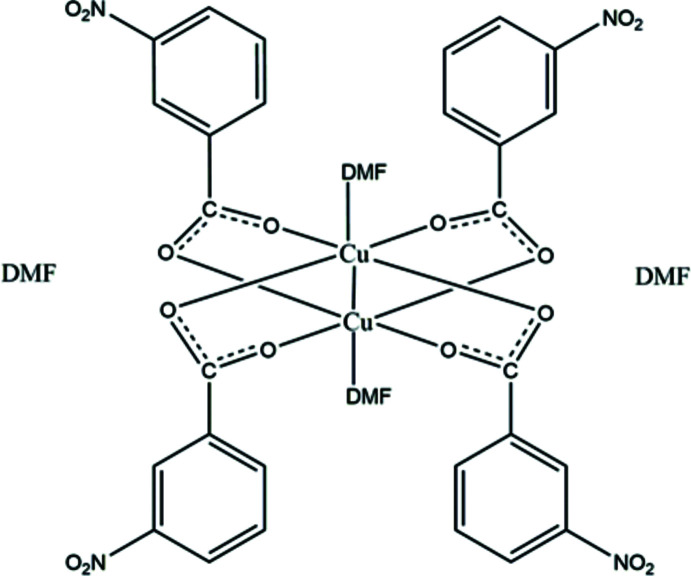


Copper(II) carboxyl­ates, including copper(II) benzoates have been studied extensively from different points of view, because the carboxyl­ato ligands exhibit different binding modes that are related to their properties, *e.g*. the basicity of the anion or the position of substituents on the aromatic ring. A bulky benzene ring substituent in an *ortho* position to the carboxyl­ate group is believed to prefer the dimeric copper(II) benzoate structure (Harrison *et al.*, 1972 ▸; Ueyama *et al.*, 1996 ▸). In general, copper(II) carboxyl­ates exhibit a dimeric paddle-wheel cage structure. More than 500 crystal structures containing the Cu_2_(OOC*R*)_4_ core have been determined on the basis of X-ray data and can be found in the Cambridge Structural Database (Groom *et al.*, 2016 ▸), of which more than 250 are of the type [Cu_2_(OOC*R*)_4_(*L*)_2_], where *L* is an apical ligand with an oxygen, a nitro­gen, a chlorine or a phospho­rus donor atom. We report here on the mol­ecular and crystal structure of a similar binuclear copper(II) complex, [Cu_2_(C_7_H_4_NO_4_)_4_(C_3_H_7_NO)_2_]·(C_3_H_7_NO)_2_, further characterized by infrared spectroscopy and DFT calculations.

## Structural commentary

**Figure 1:**
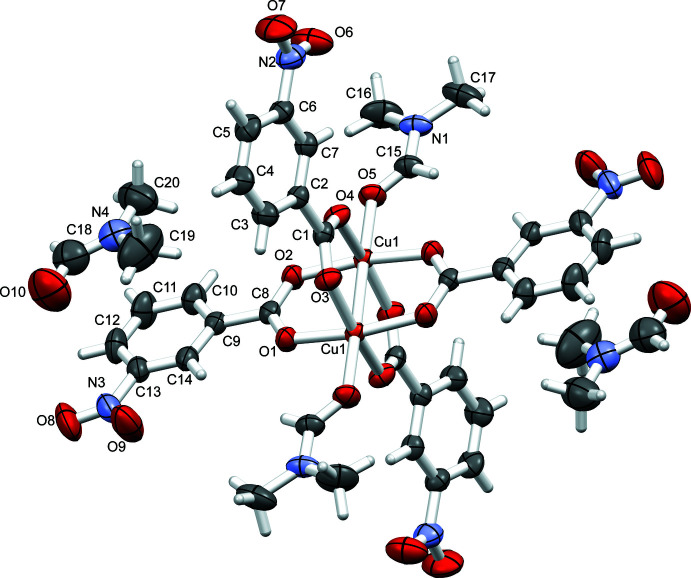
Mol­ecular structure of [Cu_2_(C_7_H_4_NO_4_)_4_(C_3_H_7_NO)_2_]·(C_3_H_7_NO)_2_, with displacement ellipsoids drawn at the 30% probability level and H atoms shown as spheres of arbitrary radius. [Symmetry code: (i) −*x* + 1, −*y* + 1, −*z* + 1]

[Cu_2_(C_7_H_4_NO_4_)_4_(C_3_H_7_NO)_2_]·(C_3_H_7_NO)_2_ crystallizes as a di­methyl­formamide disolvate (Fig. 1 ▸). The neutral [Cu_2_(C_7_H_4_NO_4_)_4_(C_3_H_7_NO)_2_] complex is centrosymmetric, with the inversion center located midway between the two Cu^II^ cations. The asymmetric unit comprises one Cu^II^ cation, two 3-nitro­benzoato ligands and two di­methyl­formamide mol­ecules, one ligating and one as a solvent. The complex displays a paddle-wheel-shaped binuclear structure. Each Cu^II^ cation is coordinated by four carboxyl­ate oxygen atoms, forming the base of a slightly distorted square pyramid supplemented by a fifth oxygen atom of the di­methyl­formamide mol­ecule at the apical position (Fig. 1 ▸). The overall distorted octa­hedral coordination environment is completed by the neighbouring Cu^II^ cation with a Cu—Cu distance of 2.6554 (6) Å. This distance is close to that reported for similar binuclear complexes (Wang *et al.*, 2018 ▸).

In the binuclear complex, the carboxyl­ate groups of the 3-nitro­benzoato ligands link the two Cu^II^ cations with short Cu—O distances [from 1.9620 (17) to 1.9751 (16) Å; Table 1 ▸] whereas the distance to the O atom of the di­methyl­formamide ligand is elongated [2.1453 (17) Å] . The carboxyl­ate groups of the 3-nitro­benzoato ligands adopt a bidentate *syn–syn* bridging mode (Su *et al.*, 2015 ▸; Wang *et al.*, 2018 ▸), with dihedral angles between the carboxyl­ate planes and the aromatic rings of 5.2 (3) and 23.9 (3)°, respectively.

## Supra­molecular features

**Figure 2:**
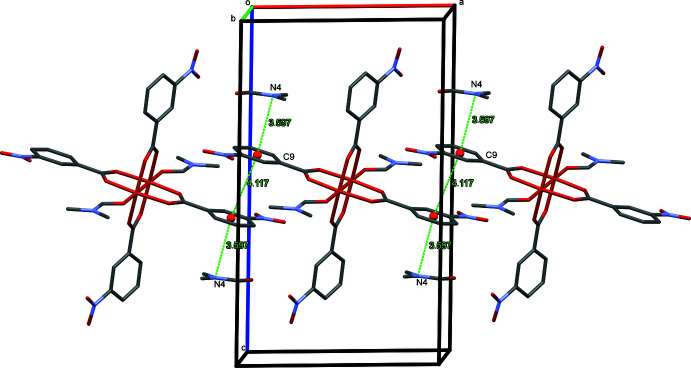
Non-aromatic–aromatic–aromatic inter­actions between adjacent binuclear metal units.

The binuclear complex mol­ecules are allocated with their central parts parallel to (200). The crystal packing shows slipped π–π stacking inter­actions between the aromatic rings of symmetry-related 3-nitro­benzoato ligands [*Cg*1⋯*Cg*1(−*x* + 2, −*y* + 1, −*z* + 1) = 4.117 (2) Å where *Cg*1 is the centroid of the C9–C14 phenyl ring; slippage 2.202 Å]. The nitro group of the second 3-nitro­benzoato ligand weakly inter­acts by O⋯C contacts [O7⋯C17(−*x* + 1, *y* + 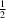 , −*z* + 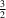 ) = 3.087 (3) Å] with the coordinating di­methyl­formamide mol­ecule, forming zigzag chains parallel to [01 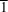 ]. Through these inter­actions, the complex mol­ecules form a channel-like structure with the channels, in which the di­methyl­formamide solvate mol­ecules are located, extending parallel to [010]. They inter­act *via* weak amide-π inter­actions [N4⋯*Cg*1 = 3.597 (3) Å] and weak C—H⋯O(nitro group) hydrogen bonds (Fig. 2 ▸, Table 2 ▸). The latter inter­actions cause a greater rotation [23.9 (3)°] of the aromatic ring relative to the carboxyl­ate group in the second 3-nitro­benzoato ligand.

## Hirshfeld surface analysis

**Figure 3:**
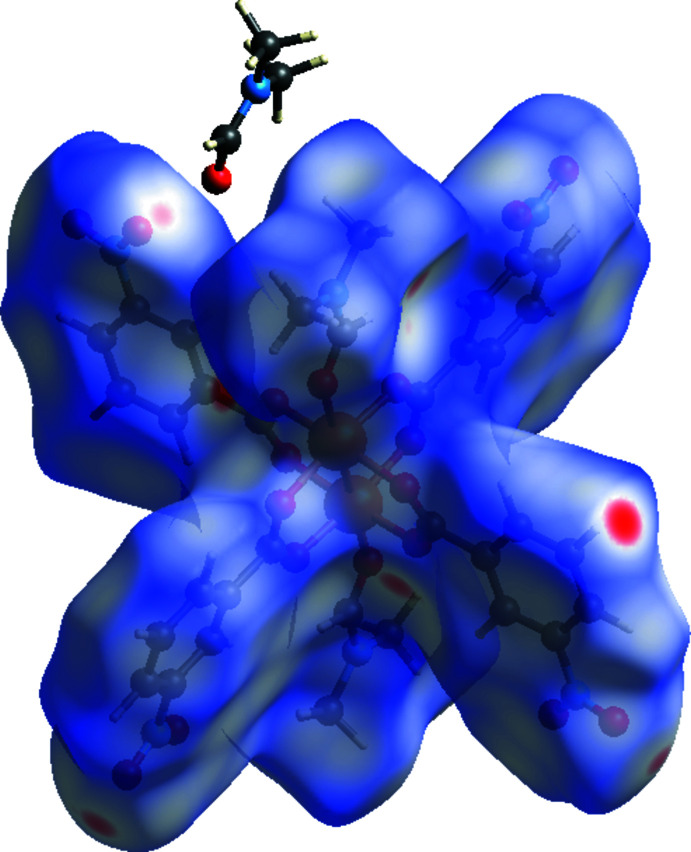
Three-dimensional Hirshfeld surface of the title compound mapped over *d*_norm_.

**Figure 4:**
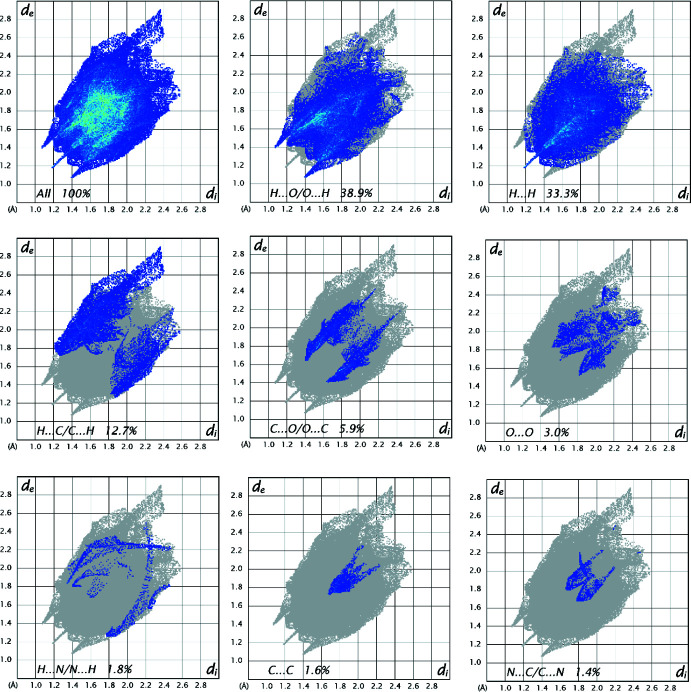
Two-dimensional fingerprint plot of [Cu_2_(C_7_H_4_NO_4_)_4_(C_3_H_7_NO)_2_]·(C_3_H_7_NO)_2_ showing all inter­actions (top left) and delineated in individual contacts with relative contributions.

Intra­molecular and inter­molecular inter­actions of [Cu_2_(C_7_H_4_NO_4_)_4_(C_3_H_7_NO)_2_]·(C_3_H_7_NO)_2_ were qu­anti­fied by Hirshfeld surface analysis using *Crystal Explorer 17.5* (Turner *et al.*, 2017 ▸). The presence of strong inter­actions on the Hirshfeld surface is indicated by red spots, while the blue areas indicate weak inter­actions, as shown in Fig. 3 ▸. Two-dimensional fingerprint plots with all inter­actions and delineated into individual inter­actions together with their relative contributions are displayed in Fig. 4 ▸. The most important inter­molecular inter­actions are O⋯H/H⋯O (38.9%), followed by H⋯H (33.3%), C⋯H/H⋯C (12.7%) and O⋯C/C⋯O (5.9%). Other inter­actions contribute less than 5% to the overall Hirshfeld surface.

## DFT calculations

Theoretical calculations were carried out by the hybrid density functional theory (DFT) at the B3LYP level of theory (Becke, 1988 ▸; Lee *et al.*, 1988 ▸) using Aldrich’s def2-TZVP basis set, which has been successfully tested in one of our previous studies (Ibragimov *et al.*, 2021 ▸). Input files for the DFT calculations using the *ORCA 4.2.0* program package (Neese, 2012 ▸) were generated by *Avogadro* (Hanwell *et al.*, 2012 ▸) using the CIF of the title compound. Results of these calculations were analyzed with the aid of *Avogadro* and *Multiwfn* (Lu & Chen, 2012 ▸).

Homonuclear Cu^II^ complexes form a closed system in which [Ar]*d* ^9^ electrons of two neighbouring Cu^II^ cations are paired with each other. Such a system is usually characterized by a singlet ground state. However, triplet and quintet electronic states are also possible, depending on the nature of the ligand mol­ecules. The bond lengths and angles of the complex were therefore fully optimized in the singlet, triplet and quintet electronic spin states with the result that the singlet electronic state was found to be the energetically optimal structure. Calculated and experimentally determined bond lengths and angles are compared in Tables S1 and S2 in the supporting information, and mean absolute errors (MAE), largest errors (LE) and the correlation coefficients *R* ^2^ were determined. The very low values of MAE and LE, and also the high *R* ^2^ coefficient of 0.997 reveal the suitability of the applied method for calculation of the electronic structure parameters of the complex.

**Figure 5:**
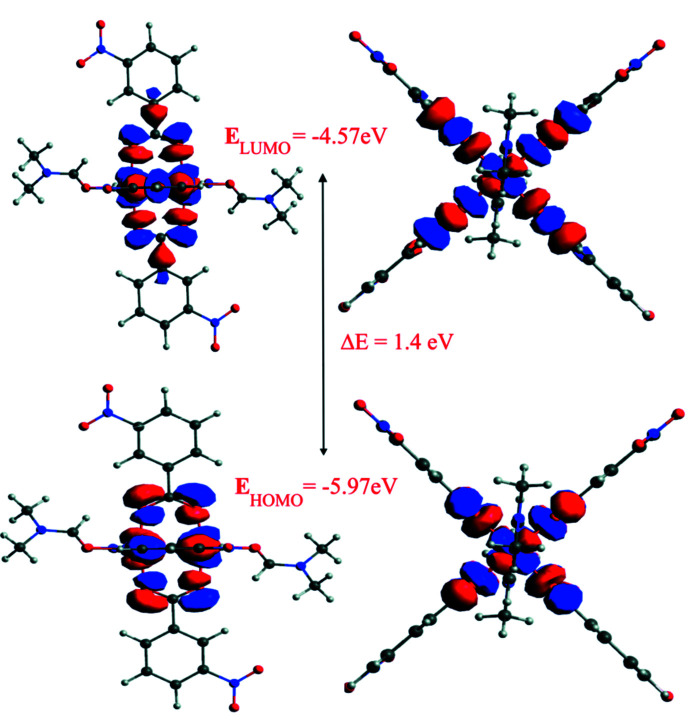
HOMO–LUMO energy diagram of [Cu_2_(C_7_H_4_NO_4_)_4_(C_3_H_7_NO)_2_]·(C_3_H_7_NO)_2_.

Calculations of electron densities on atoms in the highest occupied (HOMO) and lowest unoccupied (LUMO) mol­ecular orbitals (MO), as well as the energies of the frontier MOs (FMO) were carried out. The charge distributions on atoms and on the FMOs, as well as the energy of FMOs are one of the main parameters of the electronic structure of chemical compounds (Karelson *et al.*, 1996 ▸; Rauk, 2001 ▸; Miar *et al.*, 2021 ▸). The energy of the HOMO is related to the electron-donating ability of a mol­ecule and the energy of the LUMO is related to the electron-accepting ability of a mol­ecule. The parameter for chemical hardness (*η*) is calculated based on the HOMO–LUMO energy gap (*η* = Δ*E*/2). The shape of the FMOs and the HOMO–LUMO energy gap of the complex are displayed in Fig. 5 ▸. The contribution of both Cu^II^ *d* orbitals in the HOMO and LUMO are 58.14% and 52.72%, respectively. The contribution of the *p* orbitals of the eight oxygen atoms of the 3-nitro­benzoate ligands in the HOMO and LUMO are 37.15% and 38.06%, respectively. A higher contribution of Cu^II^ *d* electrons (56.74%) was observed in the case of second occupied MO (*E*_HOMO–1_ = −7.0 eV), and the next unoccupied MO (*E*_LUMO+1_ = −2.74 eV) consists of the anti­bonding *p* orbitals of the 3-nitro­benzoate fragment.

The complex has a very low HOMO–LUMO energy gap, which can be seen from Fig. 5 ▸ and also from the total density of state diagram (TDOS, Fig. S1) of the complex. The low energy gap is caused by a significant decreasing of the energy level of the LUMO of the complex. In other words, the electron accepting ability of the complex is very high and thus the mol­ecule becomes more susceptible to nucleophile attack which makes this complex inter­esting in chemistry and physics due to its electrical properties and light absorption at a low energy level.

Atomic charge analysis (Fig. S2) shows that the largest negative charges and the largest positive charges are located on oxygen atoms and carbon atoms of the carb­oxy­lic group, respectively.

## FT–IR analysis

**Figure 6:**
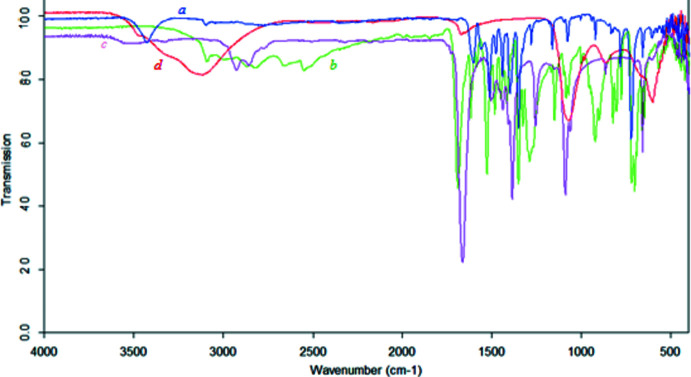
FT–IR (ATR) spectrum of [Cu_2_(C_7_H_4_NO_4_)_4_(C_3_H_7_NO)_2_]·(C_3_H_7_NO)_2_ (*a*) and the starting compounds 3-nitro­benzoic acid (*b*), di­methyl­formamide (*c*) and aqueous CuSO_4_ solution (*d*).

The FT–IR (ATR) spectrum of di­methyl­formamide (see Fig. S1) comprises the following absorption bands (cm^−1^): 2926, 2856 (–CH, NCH_3_), 2780 (C–H, CHO), 1662 (C=O), 1384 (CH, NCH_3_), 1089 (C—N). The FTIR (ATR) spectrum of 3-nitro­benzoic acid (Fig. 6 ▸) comprises the following absorption bands (cm^−1^): 3095 (C—H, Ar), 2500–3000 (OH, dimeric form), 1689 (C=O), 1614, 1583 (Ar), 1525, 1350 (–NO_2_), 1288 (C—O, COOH). The FTIR (ATR) spectrum of the title complex (Fig. 6 ▸) comprises the following absorption bands (cm^−1^): 3095 (C—H, Ar), 1600 (C=O), 1556 (Ar), 1514, 1348 (–NO_2_), 1396 (CH, NCH_3_).

Analysis of the IR spectra of the starting compounds and the product shows significant changes in the wavenumbers of absorption bands of characteristic groups in the IR spectrum of the product. Upon complexation, some absorption bands disappear, and some of them undergo a high-frequency or low-frequency shift.

For example, the C=O band of 3-nitro­benzoic (1689 cm^−1^) and di­methyl­formamide (1662 cm^−1^) shifts to the low-frequency region and is observed at 1600 cm^−1^. Likewise, the absorption band of the nitro group (1525, 1350 cm^−1^) of 3-nitro­benzoic acid is shifted to the low-frequency region (1514, 1348 cm^−1^) after complexation. At the same time, the absorption band of the –CH_3_ groups of di­methyl­formamide at 1384 cm^−1^ undergoes a high-frequency shift and is observed at 1396 cm^−1^. On the other hand, the absorption band of the C—O group at 1288 cm^−1^ of 3-nitro­benzoic acid does not occur in the IR spectrum of the complex.

## Database survey

A search of the Cambridge Structural Database (CSD, Version 2020.1 including the update of January 2020; Groom *et al.*, 2016 ▸) of binuclear copper(II) complexes comprising benzoate ligands with an *o*-nitro group gave nine hits, with an *m*-nitro group gave four hits [FAZXUA (Kabbani *et al.*, 2004 ▸), KELXEF (Stachová *et al.*, 2006 ▸), NIDSEY (Hökelek *et al.*, 1998 ▸) and PABNEP (Xu *et al.*, 2020 ▸)], and with a *p*-nitro group also gave four hits [AQNBCU (Usubaliev *et al.*, 1980 ▸), BOVPIN (Jassal *et al.*, 2015 ▸), QIXQIX01 (Li & Zhou, 2010 ▸) and VIHNAD (Song *et al.*, 2013 ▸)]. In the di­nitro­benzoate complex NIDSEY, [Cu_3_{(NO_2_)_2_C_6_H_3_COO}_6_(CH_3_-OH)_2_], comprising three copper(II) atoms, two of them are five-coordinate, being surrounded by square-pyramids of carboxyl­ate O atoms and forming a paddle-wheel-shaped binuclear structure, whereas the third copper(II) ion has a square-planar environment.

## Synthesis and crystallization

The crystals were grown from low-cost standard materials. 3-Nitro­benzoic acid (20.0 mg, 0.12 mmol) and CuSO_4_·5H_2_O (20 mg, 0.056 mmol) were mixed and stirred at room temperature for 1 h. Then, in a gradual way, di­methyl­formamide (DMF; 0.78 mmol) was added dropwise to the stirred mixture throughout 60 min at 303 K, immediately after which the solution was cooled down and kept for several hours. Darkish blue single crystals suitable for X-ray analysis were grown by slow evaporation at ambient temperature for one week and collected by filtration. They were finally washed with pure DMF. Yield: 70%.

## Refinement

Crystal data, data collection and structure refinement details are summarized in Table 3 ▸. Hydrogen atoms bonded to carbon atoms were placed in calculated positions and refined to ride on their parent atoms with C—H = 0.93 Å and *U*_iso_(H) = 1.2*U*_eq_(C) for aromatic hydrogen atoms, and with C—H = 0.96 Å and *U*_iso_(H) = 1.5*U*_eq_(C) for methyl hydrogen atoms.

## Supplementary Material

Crystal structure: contains datablock(s) I, GLOBAL. DOI: 10.1107/S2056989021010999/wm5619sup1.cif

Structure factors: contains datablock(s) I. DOI: 10.1107/S2056989021010999/wm5619Isup2.hkl

Supporting information on DFT calculations. DOI: 10.1107/S2056989021010999/wm5619sup3.docx

CCDC reference: 2036680

Additional supporting information:  crystallographic information; 3D view; checkCIF report

## Figures and Tables

**Table 1:** Selected bond lengths (Å)

Cu1—O1	1.9620 (17)	Cu1—O4^i^	1.9751 (16)
Cu1—O3	1.9650 (16)	Cu1—O5	2.1453 (17)
Cu1—O2^i^	1.9719 (18)	Cu1—Cu1^i^	2.6554 (6)

Symmetry code: (i) -x+1, -y+1, -z+1.

**Table 2:** Hydrogen-bond geometry (Å, °)

*D*—H⋯*A*	*D*—H	H⋯*A*	*D*⋯*A*	*D*—H⋯*A*
C4—H4⋯O4^ii^	0.93	2.47	3.360 (4)	161
C15—H15⋯O1	0.93	2.50	3.100 (4)	123
C16—H16*C*⋯O5	0.96	2.40	2.770 (4)	102
C19—H19*A*⋯O10	0.96	2.35	2.753 (8)	104
C20—H20*C*⋯O10^iii^	0.96	2.59	3.503 (7)	160

Symmetry codes: (ii) -x+1, y-{\script{1\over 2}}, -z+{\script{3\over 2}}; (iii) -x+2, y+{\script{1\over 2}}, -z+{\script{3\over 2}}.

**Table 3:** Experimental details

Crystal data	
Chemical formula	[Cu_2_(C_7_H_4_NO_4_)_4_(C_3_H_7_NO)_2_]·2C_3_H_7_NO
*M*_r_	1083.91
Crystal system, space group	Monoclinic, *P*2_1_/*c*
Temperature (K)	293
*a*, *b*, *c* (Å)	11.5657 (4), 10.4851 (3), 19.7258 (5)
β (°)	91.581 (3)
*V* (Å^3^)	2391.19 (12)
*Z*	2
Radiation type	Cu *K*α
μ (mm^−1^)	1.84
Crystal size (mm)	0.20 × 0.15 × 0.10

Data collection	
Diffractometer	Xcalibur, Ruby
Absorption correction	Multi-scan (*CrysAlis PRO*; Rigaku OD, 2018 ▸)
*T*_min_, *T*_max_	0.366, 1.000
No. of measured, independent and observed [*I* > 2σ(*I*)] reflections	17186, 4940, 4016
*R*_int_	0.041
(sin θ/λ)_max_ (Å^−1^)	0.630

Refinement	
*R*[*F* ^2^ > 2σ(*F* ^2^)], *wR*(*F* ^2^), *S*	0.043, 0.120, 1.05
No. of reflections	4940
No. of parameters	320
H-atom treatment	H-atom parameters constrained
Δρ_max_, Δρ_min_ (e Å^−3^)	0.38, −0.66

Computer programs: *CrysAlis PRO* (Rigaku OD, 2018 ▸), *SHELXT* (Sheldrick, 2015*a* ▸), *SHELXL2014/7* (Sheldrick, 2015*b* ▸), *Mercury* (Macrae *et al.*, 2020 ▸) and *publCIF* (Westrip, 2010 ▸).

## supplementary crystallographic information

### Crystal data

**Table:**

[Cu_2_(C_7_H_4_NO_4_)_4_(C_3_H_7_NO)_2_]·2C_3_H_7_NO	*F*(000) = 1116
*M**_r_* = 1083.91	*D*_x_ = 1.505 Mg m^−^^3^
Monoclinic, *P*2_1_/*c*	Cu *K*α radiation, λ = 1.54184 Å
*a* = 11.5657 (4) Å	Cell parameters from 5941 reflections
*b* = 10.4851 (3) Å	θ = 4.2–75.5°
*c* = 19.7258 (5) Å	µ = 1.84 mm^−^^1^
β = 91.581 (3)°	*T* = 293 K
*V* = 2391.19 (12) Å^3^	Plate, blue
*Z* = 2	0.20 × 0.15 × 0.10 mm

### Data collection

**Table:**

Xcalibur, Ruby diffractometer	4940 independent reflections
Radiation source: Enhance (Cu) X-ray Source	4016 reflections with *I* > 2σ(*I*)
Graphite monochromator	*R*_int_ = 0.041
Detector resolution: 10.2576 pixels mm^-1^	θ_max_ = 76.2°, θ_min_ = 3.8°
wσcans	*h* = −14→14
Absorption correction: multi-scan (CrysAlisPro; Rigaku OD, 2018)	*k* = −13→12
*T*_min_ = 0.366, *T*_max_ = 1.000	*l* = −24→16
17186 measured reflections	

### Refinement

**Table:**

Refinement on *F*^2^	Primary atom site location: structure-invariant direct methods
Least-squares matrix: full	Hydrogen site location: inferred from neighbouring sites
*R*[*F*^2^ > 2σ(*F*^2^)] = 0.043	H-atom parameters constrained
*wR*(*F*^2^) = 0.120	*w* = 1/[σ^2^(*F*_o_^2^) + (0.0582*P*)^2^ + 0.9153*P*] where *P* = (*F*_o_^2^ + 2*F*_c_^2^)/3
*S* = 1.05	(Δ/σ)_max_ = 0.001
4940 reflections	Δρ_max_ = 0.38 e Å^−^^3^
320 parameters	Δρ_min_ = −0.65 e Å^−^^3^
0 restraints	

### Special details

**Table:**

Geometry. All esds (except the esd in the dihedral angle between two l.s. planes) are estimated using the full covariance matrix. The cell esds are taken into account individually in the estimation of esds in distances, angles and torsion angles; correlations between esds in cell parameters are only used when they are defined by crystal symmetry. An approximate (isotropic) treatment of cell esds is used for estimating esds involving l.s. planes.

### Fractional atomic coordinates and isotropic or equivalent isotropic displacement parameters (Å^2^)

**Table:**

	*x*	*y*	*z*	*U*_iso_*/*U*_eq_	
Cu1	0.52536 (3)	0.37834 (3)	0.48912 (2)	0.03259 (12)	
O1	0.67889 (15)	0.43281 (17)	0.52255 (9)	0.0438 (4)	
O2	0.63778 (15)	0.63846 (16)	0.54126 (9)	0.0442 (4)	
O3	0.48321 (16)	0.35248 (16)	0.58396 (8)	0.0447 (4)	
O4	0.43655 (16)	0.55704 (16)	0.60163 (8)	0.0438 (4)	
O5	0.57752 (17)	0.18687 (16)	0.46685 (9)	0.0474 (4)	
O6	0.2420 (2)	0.6543 (3)	0.81319 (13)	0.0853 (8)	
O7	0.2426 (3)	0.5167 (3)	0.89371 (12)	0.0977 (10)	
O8	1.1930 (2)	0.3843 (3)	0.60860 (15)	0.0927 (9)	
O9	1.0475 (2)	0.2614 (3)	0.59089 (17)	0.1004 (10)	
N1	0.7192 (2)	0.0413 (2)	0.45278 (11)	0.0533 (6)	
N2	0.2717 (2)	0.5527 (3)	0.83778 (12)	0.0612 (7)	
N3	1.0906 (2)	0.3660 (3)	0.59792 (14)	0.0659 (8)	
C1	0.4512 (2)	0.4433 (2)	0.62026 (11)	0.0376 (5)	
C2	0.4313 (2)	0.4157 (2)	0.69403 (11)	0.0376 (5)	
C3	0.4849 (3)	0.3128 (3)	0.72534 (13)	0.0509 (7)	
H3	0.5305	0.2581	0.7004	0.061*	
C4	0.4709 (3)	0.2912 (3)	0.79400 (15)	0.0614 (8)	
H4	0.5092	0.2233	0.8150	0.074*	
C5	0.4012 (3)	0.3685 (3)	0.83118 (13)	0.0543 (7)	
H5	0.3909	0.3534	0.8771	0.065*	
C6	0.3469 (2)	0.4689 (3)	0.79880 (12)	0.0445 (6)	
C7	0.3616 (2)	0.4953 (2)	0.73090 (12)	0.0411 (6)	
H7	0.3253	0.5651	0.7105	0.049*	
C8	0.7049 (2)	0.5442 (2)	0.54082 (11)	0.0373 (5)	
C9	0.8275 (2)	0.5637 (2)	0.56579 (12)	0.0398 (5)	
C10	0.8687 (3)	0.6832 (3)	0.58268 (15)	0.0547 (7)	
H10	0.8197	0.7534	0.5794	0.066*	
C11	0.9830 (3)	0.6997 (3)	0.60457 (18)	0.0701 (9)	
H11	1.0098	0.7807	0.6161	0.084*	
C12	1.0562 (3)	0.5977 (3)	0.60927 (17)	0.0654 (9)	
H12	1.1332	0.6084	0.6231	0.078*	
C13	1.0132 (2)	0.4784 (3)	0.59303 (14)	0.0504 (7)	
C14	0.9006 (2)	0.4592 (3)	0.57205 (12)	0.0449 (6)	
H14	0.8736	0.3776	0.5622	0.054*	
C15	0.6799 (3)	0.1556 (3)	0.46790 (13)	0.0482 (6)	
H15	0.7341	0.2174	0.4803	0.058*	
C16	0.6410 (4)	−0.0611 (3)	0.4369 (2)	0.0850 (12)	
H16A	0.6331	−0.1143	0.4761	0.128*	
H16B	0.6707	−0.1107	0.4004	0.128*	
H16C	0.5668	−0.0268	0.4236	0.128*	
C17	0.8432 (3)	0.0129 (4)	0.4595 (2)	0.0837 (12)	
H17A	0.8833	0.0858	0.4779	0.126*	
H17B	0.8726	−0.0069	0.4157	0.126*	
H17C	0.8548	−0.0587	0.4892	0.126*	
O10	1.0396 (3)	0.4379 (4)	0.7733 (2)	0.1376 (14)	
N4	0.8658 (3)	0.5366 (3)	0.76200 (16)	0.0773 (8)	
C18	0.9808 (5)	0.5333 (5)	0.7716 (2)	0.0941 (13)	
H18	1.0188	0.6109	0.7774	0.113*	
C19	0.8031 (6)	0.4220 (6)	0.7556 (3)	0.145 (2)	
H19A	0.8554	0.3532	0.7469	0.217*	
H19B	0.7477	0.4292	0.7187	0.217*	
H19C	0.7636	0.4056	0.7968	0.217*	
C20	0.8067 (5)	0.6575 (5)	0.7589 (2)	0.1113 (16)	
H20A	0.7587	0.6661	0.7976	0.167*	
H20B	0.7594	0.6616	0.7182	0.167*	
H20C	0.8624	0.7253	0.7589	0.167*	

### Atomic displacement parameters (Å^2^)

**Table:**

	*U*^11^	*U*^22^	*U*^33^	*U*^12^	*U*^13^	*U*^23^
Cu1	0.0397 (2)	0.02672 (19)	0.03118 (18)	0.00108 (14)	−0.00230 (14)	−0.00110 (12)
O1	0.0408 (10)	0.0382 (9)	0.0517 (10)	0.0001 (8)	−0.0074 (8)	−0.0067 (8)
O2	0.0428 (10)	0.0384 (10)	0.0511 (10)	0.0009 (8)	−0.0070 (8)	−0.0056 (8)
O3	0.0604 (12)	0.0393 (10)	0.0346 (8)	0.0024 (8)	0.0043 (8)	0.0023 (7)
O4	0.0626 (12)	0.0360 (9)	0.0328 (8)	−0.0022 (8)	0.0026 (8)	0.0038 (7)
O5	0.0572 (12)	0.0315 (9)	0.0533 (10)	0.0070 (8)	0.0008 (9)	−0.0047 (8)
O6	0.108 (2)	0.0639 (15)	0.0859 (17)	0.0257 (15)	0.0341 (15)	0.0078 (13)
O7	0.125 (2)	0.122 (2)	0.0477 (13)	0.0305 (19)	0.0330 (14)	0.0112 (14)
O8	0.0399 (13)	0.133 (3)	0.105 (2)	0.0118 (14)	−0.0095 (13)	0.0151 (17)
O9	0.0694 (17)	0.0748 (18)	0.156 (3)	0.0204 (15)	−0.0189 (17)	−0.0202 (19)
N1	0.0728 (17)	0.0353 (12)	0.0528 (13)	0.0130 (11)	0.0181 (12)	0.0010 (10)
N2	0.0669 (17)	0.0692 (18)	0.0480 (13)	0.0005 (14)	0.0093 (12)	−0.0031 (13)
N3	0.0449 (16)	0.089 (2)	0.0635 (16)	0.0140 (15)	−0.0027 (12)	0.0034 (15)
C1	0.0405 (13)	0.0390 (13)	0.0330 (11)	−0.0051 (11)	−0.0030 (9)	0.0045 (10)
C2	0.0425 (14)	0.0361 (12)	0.0339 (11)	−0.0029 (11)	−0.0014 (10)	0.0042 (9)
C3	0.0568 (17)	0.0503 (16)	0.0456 (14)	0.0095 (14)	0.0034 (12)	0.0074 (12)
C4	0.074 (2)	0.062 (2)	0.0476 (15)	0.0153 (17)	−0.0003 (14)	0.0188 (14)
C5	0.0636 (19)	0.0632 (19)	0.0359 (13)	0.0016 (15)	0.0001 (12)	0.0138 (12)
C6	0.0498 (15)	0.0479 (15)	0.0361 (12)	−0.0027 (12)	0.0035 (11)	0.0015 (11)
C7	0.0435 (14)	0.0391 (14)	0.0404 (12)	−0.0016 (11)	−0.0025 (10)	0.0076 (10)
C8	0.0409 (13)	0.0399 (13)	0.0310 (11)	−0.0013 (11)	−0.0013 (9)	0.0010 (9)
C9	0.0402 (14)	0.0434 (14)	0.0357 (11)	−0.0029 (11)	−0.0014 (10)	−0.0003 (10)
C10	0.0547 (17)	0.0478 (16)	0.0610 (17)	−0.0100 (14)	−0.0097 (14)	0.0025 (13)
C11	0.065 (2)	0.059 (2)	0.085 (2)	−0.0241 (17)	−0.0192 (18)	0.0027 (17)
C12	0.0466 (18)	0.081 (2)	0.0672 (19)	−0.0181 (17)	−0.0165 (15)	0.0062 (17)
C13	0.0398 (14)	0.0673 (19)	0.0438 (13)	0.0021 (14)	−0.0017 (11)	0.0014 (13)
C14	0.0411 (14)	0.0527 (16)	0.0409 (13)	−0.0042 (12)	−0.0018 (11)	−0.0046 (11)
C15	0.0596 (18)	0.0360 (14)	0.0494 (14)	0.0068 (13)	0.0075 (13)	−0.0021 (11)
C16	0.110 (3)	0.0401 (18)	0.107 (3)	0.0039 (19)	0.027 (2)	−0.0158 (19)
C17	0.085 (3)	0.072 (2)	0.096 (3)	0.034 (2)	0.034 (2)	0.008 (2)
O10	0.140 (3)	0.109 (3)	0.163 (4)	0.049 (3)	−0.008 (3)	0.016 (3)
N4	0.086 (2)	0.072 (2)	0.0730 (19)	−0.0017 (18)	0.0054 (16)	0.0005 (16)
C18	0.107 (4)	0.085 (3)	0.090 (3)	0.008 (3)	0.004 (3)	0.006 (2)
C19	0.179 (6)	0.133 (5)	0.122 (4)	−0.071 (5)	0.000 (4)	−0.009 (4)
C20	0.117 (4)	0.120 (4)	0.097 (3)	0.040 (3)	0.014 (3)	0.009 (3)

### Geometric parameters (Å, º)

**Table:**

Cu1—O1	1.9620 (17)	C6—C7	1.383 (3)
Cu1—O3	1.9650 (16)	C7—H7	0.9300
Cu1—O2^i^	1.9719 (18)	C8—C9	1.502 (3)
Cu1—O4^i^	1.9751 (16)	C9—C10	1.378 (4)
Cu1—O5	2.1453 (17)	C9—C14	1.387 (4)
Cu1—Cu1^i^	2.6554 (6)	C10—C11	1.390 (4)
O1—C8	1.257 (3)	C10—H10	0.9300
O2—C8	1.257 (3)	C11—C12	1.365 (5)
O2—Cu1^i^	1.9718 (18)	C11—H11	0.9300
O3—C1	1.254 (3)	C12—C13	1.381 (4)
O4—C1	1.258 (3)	C12—H12	0.9300
O4—Cu1^i^	1.9751 (16)	C13—C14	1.371 (4)
O5—C15	1.229 (3)	C14—H14	0.9300
O6—N2	1.216 (3)	C15—H15	0.9300
O7—N2	1.222 (3)	C16—H16A	0.9600
O8—N3	1.213 (3)	C16—H16B	0.9600
O9—N3	1.211 (4)	C16—H16C	0.9600
N1—C15	1.318 (3)	C17—H17A	0.9600
N1—C16	1.432 (4)	C17—H17B	0.9600
N1—C17	1.467 (4)	C17—H17C	0.9600
N2—C6	1.469 (4)	O10—C18	1.209 (5)
N3—C13	1.481 (4)	N4—C18	1.339 (6)
C1—C2	1.508 (3)	N4—C19	1.407 (6)
C2—C3	1.381 (4)	N4—C20	1.441 (5)
C2—C7	1.381 (3)	C18—H18	0.9300
C3—C4	1.387 (4)	C19—H19A	0.9600
C3—H3	0.9300	C19—H19B	0.9600
C4—C5	1.370 (4)	C19—H19C	0.9600
C4—H4	0.9300	C20—H20A	0.9600
C5—C6	1.374 (4)	C20—H20B	0.9600
C5—H5	0.9300	C20—H20C	0.9600
			
O1—Cu1—O3	88.06 (8)	O1—C8—C9	115.9 (2)
O1—Cu1—O2^i^	167.89 (7)	C10—C9—C14	119.4 (2)
O3—Cu1—O2^i^	90.95 (8)	C10—C9—C8	121.3 (2)
O1—Cu1—O4^i^	88.98 (8)	C14—C9—C8	119.3 (2)
O3—Cu1—O4^i^	167.85 (7)	C9—C10—C11	120.5 (3)
O2^i^—Cu1—O4^i^	89.47 (8)	C9—C10—H10	119.7
O1—Cu1—O5	94.83 (7)	C11—C10—H10	119.7
O3—Cu1—O5	98.30 (7)	C12—C11—C10	120.4 (3)
O2^i^—Cu1—O5	97.26 (7)	C12—C11—H11	119.8
O4^i^—Cu1—O5	93.69 (7)	C10—C11—H11	119.8
O1—Cu1—Cu1^i^	82.41 (5)	C11—C12—C13	118.4 (3)
O3—Cu1—Cu1^i^	85.24 (5)	C11—C12—H12	120.8
O2^i^—Cu1—Cu1^i^	85.48 (5)	C13—C12—H12	120.8
O4^i^—Cu1—Cu1^i^	82.68 (5)	C14—C13—C12	122.4 (3)
O5—Cu1—Cu1^i^	175.46 (6)	C14—C13—N3	118.0 (3)
C8—O1—Cu1	125.04 (17)	C12—C13—N3	119.5 (3)
C8—O2—Cu1^i^	120.92 (16)	C13—C14—C9	118.9 (3)
C1—O3—Cu1	121.59 (15)	C13—C14—H14	120.6
C1—O4—Cu1^i^	123.95 (16)	C9—C14—H14	120.6
C15—O5—Cu1	121.53 (18)	O5—C15—N1	125.2 (3)
C15—N1—C16	120.8 (3)	O5—C15—H15	117.4
C15—N1—C17	120.4 (3)	N1—C15—H15	117.4
C16—N1—C17	118.5 (3)	N1—C16—H16A	109.5
O6—N2—O7	123.3 (3)	N1—C16—H16B	109.5
O6—N2—C6	118.7 (2)	H16A—C16—H16B	109.5
O7—N2—C6	118.0 (3)	N1—C16—H16C	109.5
O9—N3—O8	124.1 (3)	H16A—C16—H16C	109.5
O9—N3—C13	117.9 (3)	H16B—C16—H16C	109.5
O8—N3—C13	118.1 (3)	N1—C17—H17A	109.5
O3—C1—O4	126.4 (2)	N1—C17—H17B	109.5
O3—C1—C2	117.4 (2)	H17A—C17—H17B	109.5
O4—C1—C2	116.2 (2)	N1—C17—H17C	109.5
C3—C2—C7	119.8 (2)	H17A—C17—H17C	109.5
C3—C2—C1	120.2 (2)	H17B—C17—H17C	109.5
C7—C2—C1	119.9 (2)	C18—N4—C19	119.9 (4)
C2—C3—C4	120.1 (3)	C18—N4—C20	119.8 (4)
C2—C3—H3	120.0	C19—N4—C20	120.3 (4)
C4—C3—H3	120.0	O10—C18—N4	125.5 (5)
C5—C4—C3	120.8 (3)	O10—C18—H18	117.3
C5—C4—H4	119.6	N4—C18—H18	117.3
C3—C4—H4	119.6	N4—C19—H19A	109.5
C4—C5—C6	118.2 (2)	N4—C19—H19B	109.5
C4—C5—H5	120.9	H19A—C19—H19B	109.5
C6—C5—H5	120.9	N4—C19—H19C	109.5
C5—C6—C7	122.5 (3)	H19A—C19—H19C	109.5
C5—C6—N2	119.0 (2)	H19B—C19—H19C	109.5
C7—C6—N2	118.5 (2)	N4—C20—H20A	109.5
C2—C7—C6	118.6 (2)	N4—C20—H20B	109.5
C2—C7—H7	120.7	H20A—C20—H20B	109.5
C6—C7—H7	120.7	N4—C20—H20C	109.5
O2—C8—O1	126.1 (2)	H20A—C20—H20C	109.5
O2—C8—C9	118.0 (2)	H20B—C20—H20C	109.5
			
Cu1—O3—C1—O4	3.6 (4)	Cu1—O1—C8—C9	179.02 (15)
Cu1—O3—C1—C2	−174.57 (15)	O2—C8—C9—C10	−6.0 (4)
Cu1^i^—O4—C1—O3	−5.6 (4)	O1—C8—C9—C10	175.3 (2)
Cu1^i^—O4—C1—C2	172.56 (15)	O2—C8—C9—C14	173.9 (2)
O3—C1—C2—C3	23.2 (4)	O1—C8—C9—C14	−4.8 (3)
O4—C1—C2—C3	−155.2 (3)	C14—C9—C10—C11	1.2 (4)
O3—C1—C2—C7	−158.5 (2)	C8—C9—C10—C11	−178.9 (3)
O4—C1—C2—C7	23.1 (3)	C9—C10—C11—C12	0.4 (5)
C7—C2—C3—C4	−1.4 (4)	C10—C11—C12—C13	−1.2 (5)
C1—C2—C3—C4	176.9 (3)	C11—C12—C13—C14	0.5 (5)
C2—C3—C4—C5	2.0 (5)	C11—C12—C13—N3	−179.8 (3)
C3—C4—C5—C6	−0.8 (5)	O9—N3—C13—C14	−8.5 (4)
C4—C5—C6—C7	−1.0 (4)	O8—N3—C13—C14	171.4 (3)
C4—C5—C6—N2	179.9 (3)	O9—N3—C13—C12	171.8 (3)
O6—N2—C6—C5	165.9 (3)	O8—N3—C13—C12	−8.4 (4)
O7—N2—C6—C5	−13.7 (4)	C12—C13—C14—C9	1.1 (4)
O6—N2—C6—C7	−13.2 (4)	N3—C13—C14—C9	−178.7 (2)
O7—N2—C6—C7	167.2 (3)	C10—C9—C14—C13	−1.9 (4)
C3—C2—C7—C6	−0.4 (4)	C8—C9—C14—C13	178.2 (2)
C1—C2—C7—C6	−178.7 (2)	Cu1—O5—C15—N1	−177.7 (2)
C5—C6—C7—C2	1.6 (4)	C16—N1—C15—O5	−3.6 (4)
N2—C6—C7—C2	−179.3 (2)	C17—N1—C15—O5	−176.6 (3)
Cu1^i^—O2—C8—O1	−0.5 (3)	C19—N4—C18—O10	2.5 (7)
Cu1^i^—O2—C8—C9	−178.98 (15)	C20—N4—C18—O10	−178.0 (4)
Cu1—O1—C8—O2	0.5 (4)		

Symmetry code: (i) −*x*+1, −*y*+1, −*z*+1.

### Hydrogen-bond geometry (Å, º)

**Table:**

*D*—H···*A*	*D*—H	H···*A*	*D*···*A*	*D*—H···*A*
C4—H4···O4^ii^	0.93	2.47	3.360 (4)	161
C15—H15···O1	0.93	2.50	3.100 (4)	123
C16—H16*C*···O5	0.96	2.40	2.770 (4)	102
C19—H19*A*···O10	0.96	2.35	2.753 (8)	104
C20—H20*C*···O10^iii^	0.96	2.59	3.503 (7)	160

Symmetry codes: (ii) −*x*+1, *y*−1/2, −*z*+3/2; (iii) −*x*+2, *y*+1/2, −*z*+3/2.
