# Supplementary material for: Crystal structure, Hirshfeld surface analysis and DFT studies of tetra­kis­(μ-3-nitro­benzoato-κ2 O 1:O 1′)bis­[(N,N-di­methyl­formamide-κO)copper(II)] di­methyl­formamide disolvate
Source: Acta Crystallogr E Crystallogr Commun. 2021 Oct 26;77(Pt 11):1164–9. doi: 10.1107/S2056989021010999 (PMC8587988; doi:10.1107/S2056989021010999)
Supplement: Supplementary file 3 [file e-77-01164-sup3.docx]

| Table S1. Comparison of experimental and theoretical bond lengths | | | | | | |
| --- | --- | --- | --- | --- | --- | --- |
| Number | Atom1 | Atom2 | Cyclicity | XRLength | DFT length | difference |
| 1 | Cu1 | O1 | cyclic | 1.962 | 2.019 | 0.057 |
| 2 | Cu1 | O3 | cyclic | 1.965 | 2.005 | 0.04 |
| 3 | Cu1 | O5 | acyclic | 2.145 | 2.235 | 0.09 |
| 4 | Cu1 | Cu1 | cyclic | 2.6554 | 2.644 | 0.0114 |
| 5 | Cu1 | O2 | cyclic | 1.971 | 2.004 | 0.033 |
| 6 | Cu1 | O4 | cyclic | 1.975 | 2.019 | 0.044 |
| 7 | O1 | C8 | cyclic | 1.257 | 1.259 | 0.002 |
| 8 | O2 | C8 | cyclic | 1.257 | 1.257 | 0 |
| 9 | O2 | Cu1 | cyclic | 1.971 | 2.004 | 0.033 |
| 10 | O3 | C1 | cyclic | 1.253 | 1.253 | 0 |
| 11 | O4 | C1 | cyclic | 1.258 | 1.259 | 0.001 |
| 12 | O4 | Cu1 | cyclic | 1.975 | 2.019 | 0.044 |
| 13 | O5 | C15 | acyclic | 1.229 | 1.23 | 0.001 |
| 14 | O6 | N2 | acyclic | 1.215 | 1.222 | 0.007 |
| 15 | O7 | N2 | acyclic | 1.223 | 1.221 | 0.002 |
| 16 | O8 | N3 | acyclic | 1.212 | 1.221 | 0.009 |
| 17 | O9 | N3 | acyclic | 1.211 | 1.222 | 0.011 |
| 18 | N1 | C15 | acyclic | 1.318 | 1.342 | 0.024 |
| 19 | N1 | C16 | acyclic | 1.433 | 1.453 | 0.02 |
| 20 | N1 | C17 | acyclic | 1.465 | 1.452 | 0.013 |
| 21 | N2 | C6 | acyclic | 1.468 | 1.477 | 0.009 |
| 22 | N3 | C13 | acyclic | 1.481 | 1.477 | 0.004 |
| 23 | C1 | C2 | acyclic | 1.508 | 1.508 | 0 |
| 24 | C2 | C3 | cyclic | 1.381 | 1.396 | 0.015 |
| 25 | C2 | C7 | cyclic | 1.381 | 1.392 | 0.011 |
| 26 | C3 | C4 | cyclic | 1.388 | 1.39 | 0.002 |
| 27 | C4 | C5 | cyclic | 1.369 | 1.388 | 0.019 |
| 28 | C5 | C6 | cyclic | 1.374 | 1.389 | 0.015 |
| 29 | C6 | C7 | cyclic | 1.383 | 1.385 | 0.002 |
| 30 | C8 | C9 | acyclic | 1.502 | 1.509 | 0.007 |
| 31 | C9 | C10 | cyclic | 1.378 | 1.396 | 0.018 |
| 32 | C9 | C14 | cyclic | 1.387 | 1.392 | 0.005 |
| 33 | C10 | C11 | cyclic | 1.392 | 1.39 | 0.002 |
| 34 | C11 | C12 | cyclic | 1.365 | 1.388 | 0.023 |
| 35 | C12 | C13 | cyclic | 1.379 | 1.389 | 0.01 |
| 36 | C13 | C14 | cyclic | 1.372 | 1.385 | 0.013 |
| 37 | Cu1 | O1 | cyclic | 1.962 | 2.019 | 0.057 |
| 38 | Cu1 | O3 | cyclic | 1.965 | 2.005 | 0.04 |
| 39 | Cu1 | O5 | acyclic | 2.145 | 2.235 | 0.09 |
| 40 | O1 | C8 | cyclic | 1.257 | 1.259 | 0.002 |
| 41 | O2 | C8 | cyclic | 1.257 | 1.257 | 0 |
| 42 | O3 | C1 | cyclic | 1.253 | 1.253 | 0 |
| 43 | O4 | C1 | cyclic | 1.258 | 1.259 | 0.001 |
| 44 | O5 | C15 | acyclic | 1.229 | 1.23 | 0.001 |
| 45 | O6 | N2 | acyclic | 1.215 | 1.222 | 0.007 |
| 46 | O7 | N2 | acyclic | 1.223 | 1.221 | 0.002 |
| 47 | O8 | N3 | acyclic | 1.212 | 1.221 | 0.009 |
| 48 | O9 | N3 | acyclic | 1.211 | 1.222 | 0.011 |
| 49 | N1 | C15 | acyclic | 1.318 | 1.342 | 0.024 |
| 50 | N1 | C16 | acyclic | 1.433 | 1.453 | 0.02 |
| 51 | N1 | C17 | acyclic | 1.465 | 1.452 | 0.013 |
| 52 | N2 | C6 | acyclic | 1.468 | 1.477 | 0.009 |
| 53 | N3 | C13 | acyclic | 1.481 | 1.477 | 0.004 |
| 54 | C1 | C2 | acyclic | 1.508 | 1.508 | 0 |
| 55 | C2 | C3 | cyclic | 1.381 | 1.396 | 0.015 |
| 56 | C2 | C7 | cyclic | 1.381 | 1.392 | 0.011 |
| 57 | C3 | C4 | cyclic | 1.388 | 1.39 | 0.002 |
| 58 | C4 | C5 | cyclic | 1.369 | 1.388 | 0.019 |
| 59 | C5 | C6 | cyclic | 1.374 | 1.389 | 0.015 |
| 60 | C6 | C7 | cyclic | 1.383 | 1.385 | 0.002 |
| 61 | C8 | C9 | acyclic | 1.502 | 1.509 | 0.007 |
| 62 | C9 | C10 | cyclic | 1.378 | 1.396 | 0.018 |
| 63 | C9 | C14 | cyclic | 1.387 | 1.392 | 0.005 |
| 64 | C10 | C11 | cyclic | 1.392 | 1.39 | 0.002 |
| 65 | C11 | C12 | cyclic | 1.365 | 1.388 | 0.023 |
| 66 | C12 | C13 | cyclic | 1.379 | 1.389 | 0.01 |
| 67 | C13 | C14 | cyclic | 1.372 | 1.385 | 0.013 |
| total |  |  |  |  |  | 1.0294 |
|  |  |  |  |  | MAE | 0.015364 |
|  |  |  |  |  | R^2 | 0.9972 |

| Table S2. Comparison of experimental and theoretical bond angles | | | | | | |
| --- | --- | --- | --- | --- | --- | --- |
| Number | Atom1 | Atom2 | Atom3 | Angle XRY | Angle DFT | difference |
| 1 | O1 | Cu1 | O5 | 94.83 | 93.1 | 1.73 |
| 2 | O1 | Cu1 | Cu1 | 82.41 | 83.8 | 1.39 |
| 3 | O1 | Cu1 | O2 | 167.88 | 168.3 | 0.42 |
| 4 | O1 | Cu1 | O4 | 88.99 | 88.2 | 0.79 |
| 5 | O3 | Cu1 | O5 | 98.32 | 98.4 | 0.08 |
| 6 | O3 | Cu1 | Cu1 | 85.23 | 84.2 | 1.03 |
| 7 | O3 | Cu1 | O2 | 90.94 | 90 | 0.94 |
| 8 | O3 | Cu1 | O4 | 167.83 | 168.3 | 0.47 |
| 9 | O5 | Cu1 | Cu1 | 175.46 | 175.9 | 0.44 |
| 10 | Cu1 | O1 | C8 | 125.1 | 122.2 | 2.9 |
| 11 | C8 | O2 | Cu1 | 120.9 | 122 | 1.1 |
| 12 | C1 | O4 | Cu1 | 123.94 | 121.9 | 2.04 |
| 13 | Cu1 | O5 | C15 | 121.6 | 120.1 | 1.5 |
| 14 | C15 | N1 | C16 | 120.7 | 121 | 0.3 |
| 15 | C15 | N1 | C17 | 120.47 | 121.4 | 0.93 |
| 16 | C16 | N1 | C17 | 118.47 | 117.5 | 0.97 |
| 17 | O6 | N2 | O7 | 123.31 | 124.6 | 1.29 |
| 18 | O7 | N2 | C6 | 117.9 | 117.6 | 0.3 |
| 19 | O8 | N3 | O9 | 124.07 | 124.6 | 0.53 |
| 20 | O8 | N3 | C13 | 118.06 | 117.7 | 0.36 |
| 21 | O9 | N3 | C13 | 117.9 | 117.8 | 0.1 |
| 22 | O3 | C1 | O4 | 126.36 | 127.3 | 0.94 |
| 23 | O4 | C1 | C2 | 116.2 | 116.4 | 0.2 |
| 24 | C1 | C2 | C3 | 120.2 | 120.4 | 0.2 |
| 25 | C3 | C2 | C7 | 119.84 | 119.7 | 0.14 |
| 26 | C3 | C4 | C5 | 120.7 | 120.4 | 0.3 |
| 27 | C4 | C5 | C6 | 118.27 | 118.4 | 0.13 |
| 28 | C5 | C6 | C7 | 122.43 | 122.2 | 0.23 |
| 29 | O1 | C8 | O2 | 126.12 | 127.3 | 1.18 |
| 30 | O2 | C8 | C9 | 117.9 | 116.3 | 1.6 |
| 31 | C10 | C9 | C14 | 119.4 | 119.7 | 0.3 |
| 32 | C9 | C10 | C11 | 120.5 | 120.4 | 0.1 |
| 33 | N3 | C13 | C12 | 119.57 | 119 | 0.57 |
| 34 | C12 | C13 | C14 | 122.45 | 122.2 | 0.25 |
| 35 | O5 | C15 | N1 | 125.3 | 124.9 | 0.4 |
| 36 | Cu1 | Cu1 | O2 | 85.47 | 84.5 | 0.97 |
| 37 | Cu1 | Cu1 | O4 | 82.69 | 84.1 | 1.41 |
| 38 | O2 | Cu1 | O4 | 89.48 | 89.7 | 0.22 |
| 39 | O2 | Cu1 | O5 | 97.26 | 98.6 | 1.34 |
| 40 | O4 | Cu1 | O5 | 93.69 | 93.2 | 0.49 |
| 41 | O1 | Cu1 | O3 | 88.05 | 89.7 | 1.65 |
| 42 | Cu1 | O3 | C1 | 121.6 | 122.5 | 0.9 |
| 43 | O6 | N2 | C6 | 118.74 | 117.8 | 0.94 |
| 44 | O3 | C1 | C2 | 117.4 | 116.3 | 1.1 |
| 45 | C1 | C2 | C7 | 119.94 | 119.8 | 0.14 |
| 46 | C2 | C3 | C4 | 120.1 | 120.4 | 0.3 |
| 47 | N2 | C6 | C5 | 119.04 | 119 | 0.04 |
| 48 | N2 | C6 | C7 | 118.52 | 118.8 | 0.28 |
| 49 | C2 | C7 | C6 | 118.56 | 118.9 | 0.34 |
| 50 | O1 | C8 | C9 | 115.9 | 116.4 | 0.5 |
| 51 | C8 | C9 | C10 | 121.32 | 120.4 | 0.92 |
| 52 | C8 | C9 | C14 | 119.27 | 119.8 | 0.53 |
| 53 | C10 | C11 | C12 | 120.36 | 120.4 | 0.04 |
| 54 | C11 | C12 | C13 | 118.44 | 118.4 | 0.04 |
| 55 | N3 | C13 | C14 | 117.98 | 118.8 | 0.82 |
| 56 | C9 | C14 | C13 | 118.8 | 118.9 | 0.1 |
| total |  |  |  |  |  | 39.22 |
|  |  |  |  |  | MAE | 0.700357 |
|  |  |  |  |  |  | R² = 0.9975 |


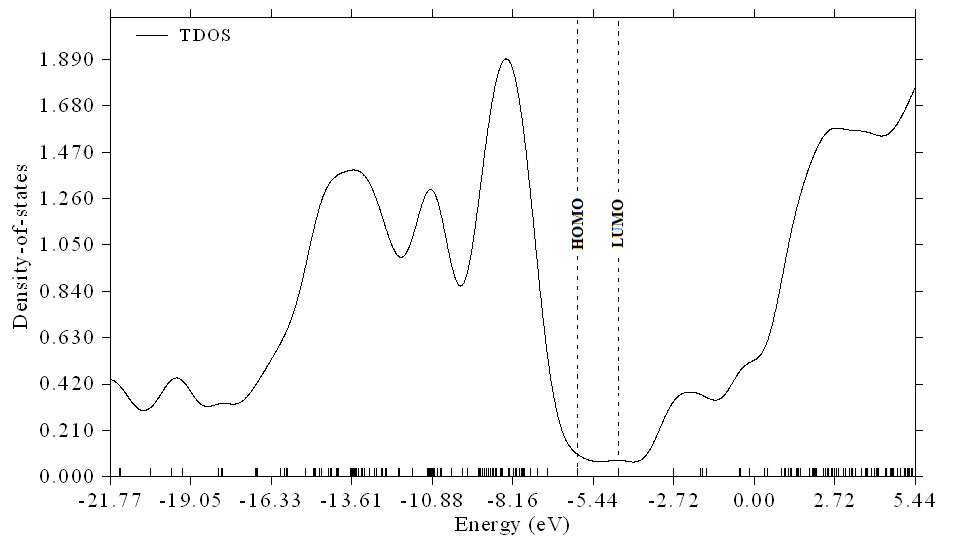


Fig.S1. Total density of states of the complex.


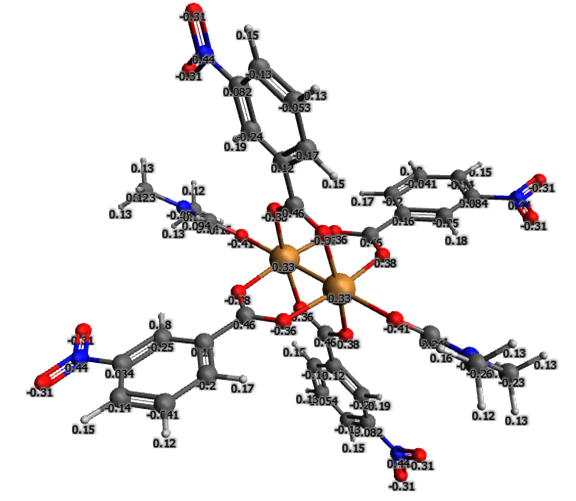


Fig.S2. Mulliken atomic charges of the complex
